# Stearic acid induces CCK and GLP-1 upregulation *via* GPR120/PLC-β, leading to reduced appetite in Hu sheep fed with rice straw

**DOI:** 10.3389/fvets.2022.948074

**Published:** 2022-09-02

**Authors:** Xi Chen, Xintian Nie, Huanhuan Wang, Shuping Yan, Yuanshu Zhang

**Affiliations:** ^1^Key Laboratory of Animal Physiology and Biochemistry, Ministry of Agriculture, College of Veterinary Medicine, Nanjing Agricultural University, Nanjing, China; ^2^College of Engineering, Nanjing Agricultural University, Nanjing, China

**Keywords:** rice straw, glucagon-like peptide-1, cholecystokinin, metabolomics, stearic acid, GPR120

## Abstract

Due to the poor palatability of straw, feeding untreated rice straw reduces ruminant feed intake, thus affecting the production efficiency of animal husbandry. However, the detailed mechanism by which straw affects ruminants' feed intake is unclear. Therefore, this study aimed to elucidate the molecular mechanism by which a rice straw (RS)-based diet affects appetite regulation in Hu sheep. We found that RS promoted the secretion of cholecystokinin (CCK) and glucagon-like peptide-1 (GLP-1) and decreased feed intake. Blood metabolomics showed that RS activated the arachidonic acid metabolism, biosynthesis of unsaturated fatty acids, linoleic acid metabolism, and alpha-linolenic acid metabolism pathways, and the secretion of stearic acid (SA), their metabolic end product, increased significantly. GPR120, one of the classical receptors of long-chain fatty acids (LCFAs), can be involved in appetite regulation. However, the role of SA in satiety hormone regulation mediated by GPR120 in ruminants is unclear. In this study, *in vivo* experiments showed that in sheep fed with RS, SA increased significantly and activated GPR120/Ca^2+^, increasing the secretion of the satiety hormones CCK and GLP-1. In vitro mechanism studies showed that SA promotes GLP-1 and CCK secretion by activating GPR120-mediated downstream PKC and IP3R signaling pathways of PLCβ.

## Introduction

The health and performance of ruminants are affected by the quality of roughage, which ultimately affects the production efficiency of animal husbandry (1). Alfalfa hay (AH) represents most of the cost of ruminant diet. To reduce the cost, it is necessary to find alternative, inexpensive feeds, such as crop residue, straw stalk. Study have reported that expander–extruder pelleting of complete diets containing red gram straw increases feed intake and weight in goats (2). Compared with rice straw pretreated with urea plus manure, feeding RS reduced the dry matter intake (DMI) of goats by 17 g/day (3). In conclusion, feed intake of ruminants decreased when untreated straw, especially rice straw, was used as roughage. However, the specific mechanism by which RS reduces feed intake in ruminants remains unclear.

The unique rumen of ruminants can decompose fiber to produce free fatty acids (FFAs), which are eventually absorbed and utilized by the intestine (4), further activating enteroendocrine cells (EECs). EEC expression includes a series of sensory proteins, such as G protein-coupled receptors (GPCRs), transporters, and ion channels through which nutrients such as FFAs trigger cascade reactions that ultimately release gut hormones such as glucagon-like peptide-1 (GLP-1) and cholecystokinin (CCK) (5). For example, L-phenylalanine induces the secretion of CCK in porcine duodenum by activating the IP3 receptor (IP3R) and protein kinase C (PKC) signaling pathways triggered by calcium-sensing receptor (6). Succinate and maleate-induced CCK release can be inhibited by blocking calcium channels (7). Corn oil induces CCK secretion through the long-chain fatty acid receptor GPR120 in mice (8). GPR120 can also increase GLP-1 secretion through phospholipase Cβ (PLCβ) and intracellular calcium ion (Ca^2+^) signaling in mice and humans (9). While most gut hormones have been shown to regulate feed intake, GLP-1 and CCK are known to be associated with anorexia (10). GLP-1, released by intestinal endocrine L cells, slows gastric emptying (11). CCK, released by intestinal endocrine I cells, has also been found to increase satiety and reduce body weight in rats (12) and humans (13).

It is currently unknown whether ruminant appetite regulation is similar. Therefore, this study aimed to analyse the satiety changes in Hu sheep fed with RS, screen out the key differential metabolites using blood metabolomics, explore the key metabolic pathways using bioinformatics analyses, and elucidate the molecular mechanism by which RS feeding affects appetite regulation in Hu sheep.

## Materials and methods

### Animals, experimental design, and diets

Twelve healthy adult male Hu sheep [mean body weight ± standard error of the mean (SEM): 41.25 ± 3.07 kg] aged 10–14 months (Xinnong Hu sheep–breeding factory, China) were randomly divided into two groups. The diet of AH group was TMR with alfalfa hay as forage (40:60, forage: concentrate ratio; *n* = 6), the diet of RS group was TMR with rice straw as forage (40:60, forage: concentrate ratio; *n* = 6) (Supplementary Table S1). The changes in feed intake and body weight over a period of 28 days were recorded. The feed conversion ratio (FCR) was calculated according to the method of Sinpru (14).

All animals were kept in a pathogen-free environment and fed ad lib. The procedures for care and use of animals were approved by the Ethics Committee of Nanjing Agricultural University and all applicable institutional and governmental regulations concerning the ethical use of animals were followed.

### Sample collection

On the last day of the experimental period, the 12 sheep were sacrificed by a registered veterinarian using jugular bleeding after a 16-h fast. Before sacrifice, 10 mL of blood was collected from the jugular vein into a heparinized syringe and immediately centrifuged at 2,500 × g at 4 °C for 15 min. The plasma was then collected into 2-mL centrifuge tubes. Plasma samples were sent to the Jiangsu Academy of Agricultural Sciences for fatty acid content analysis and to BGI Genomics Co., Ltd. for metabolomic analysis. Jejunal tissue samples (100 mg) were lysed with 1 mL of lysis buffer and fully homogenized using a homogenizer. The supernatant fluid was centrifuged at 6,000 r/min for 20 min and carefully collected.

### Metabolomic fingerprinting analysis

UPLC-MS was used to obtain the entire metabolite fingerprint of serum samples. Chromatographic separations were performed on a UPLC system (2777C, Waters, UK) and an ACQUITY HSS T3 UPLC column (100 mm × 2.1 mm, 1.8 μm; Waters, UK). The small molecules eluted from the column were collected in positive and negative ion modes using a high-resolution tandem MS system (Xevo G2-XS QTOF; Waters, UK). In positive ion mode, the capillary voltage and cone voltage were 3 kV and 40 V, respectively. In negative ion mode, they were 2 kV and 40 V, respectively. The first-level scan range was 50–1,200 Da, and the scan time was 0.2 s. All precursor ions were fragmented according to an energy of 20–40 eV, and all fragment information was collected. The scan time was 0.2 s. During data acquisition, real-time quality correction was performed on the LE signal every 3 s. Local polynomial regression fitting correction of real sample signals was performed based on the quality control sample information. Quality control sample–based robust LOESS signal correction was performed according to a previously reported method (15).

### Multivariate metabolomic analysis

A Xevo G2-XS QTOF (Waters, UK) system was used for MS data acquisition. Commercial software Progenesis QI version 2.2 (Waters, UK) and the independently developed metabolomics R package metaX (16) were used for MS data statistical analysis. Metabolite identification was performed using the HMDB (http://www.hmdb.ca/). The VIP (Variable Importance in Projection) values of the first two principal components of a multivariate partial least squares–discriminant analysis (PLS-DA) model were adopted, and the fold change and q values were combined to screen differentially expressed metabolites. The screening criteria were as follows: (1) VIP ≥ 1; (2) fold change ≥ 2 or ≤ 0.5; (3) *P* values < 0.01.

### Metabolic pathway analysis

Compounds with accurate mass-to-charge ratios were annotated using the HMDB (http://www.hmdb.ca/) and KEGG (http://www.genome.jp/kegg/). Network reconstruction and pathway analysis were performed using metabolite set enrichment analysis. The Metscape plug-in in Cytoscape version 3.6.0 was used to visualize the relationships between important metabolites.

### Cell culture

STC-1 cells, an intestinal EEC line, were procured from the Cell Bank of the Chinese Academy of Sciences (BNCC338701). The cells were cultured in Dulbecco's Modified Eagle Medium (Corning, USA) with 10% fetal bovine serum (HyClone, USA) and 1% penicillin/streptomycin (Invitrogen, USA) containing 5% CO_2_ at 37 °C. After 80–90% of confluent cells were digested with a 0.25% trypsin−0.04% EDTA solution (HyClone, USA), they were subcultured in a fresh medium in 6- or 96-well plates.

### Cell viability assay

Cell Counting Kit-8 (CCK-8) assays (APExBIO Technology LLC, USA) were performed to estimate STC-1 cell viability according to the manufacturer's instructions. In brief, cells were seeded in a 96-well plate at a density of 5 × 104 cells/well and cultured overnight. The STC-1 cells were co-cultured with SA (W303518-1KG-K, Sigma-Aldrich, USA), GW9508 (T1781, Topscience Co. Ltd, China), AH7614 (T22027), nitrendipine (T0119) or 2-APB (T4693) in 96-well culture plates (Corning Inc., USA), and 10 μL of CCK-8 reagent was added to each well for 2 h. A Synergy H1 spectrophotometer (Biotek, USA) was used to measure the optical density at 450 nm. Group settings were consistent with the description of Co-Culture.

### Real-time quantitative reverse transcription-polymerase chain reaction

Total RNA was isolated using a TRIzol reagent kit (Vazyme, China) according to the manufacturer's instructions. To investigate mRNA expression, cDNA was synthesized with a Prime Script™ II 1st strand cDNA Synthesis Kit (Takara, Japan). All samples were examined in triplicate and programmed to conduct one cycle (95 °C for 5 min) and 40 cycles (95 °C for 10 s, 60 °C for 30 s, 72 °C for 30 s). The dissolution curves were generated according to the following procedures: 95 °C/15 s, 60 °C/1 min, and 95 °C/15 s. GAPDH was used as an internal reference for normalization. SYBR Green PCR Mix (Takara, Japan) was used for quantitative analysis on a QuantStudio 12K Flex Real-Time PCR System (Applied Biosystems, USA). The relative mRNA expression levels were calculated using the 2^−Δ*ΔCt*^ method. The primers for RT-qPCR are listed in Supplementary Table S2.

### Determination of Ca^2+^, GLP-1, and CCK levels

The Ca^2+^ concentrations in jejunum tissues were determined using Calcium Assay Kit according to the manufacturer's instructions (Jiancheng, China). The absorbance (optical density value) of each well was measured at a wavelength of 450 nm, and the Ca^2+^ content was calculated according to the standard curve and expressed as mmol/g. GLP-1 (MM-50903O1, Jiangsu enzyme industry Co., Ltd, China) and CCK (MM-1488O1) concentrations in plasma were determined by enzyme linked immunosorbent assay (ELISA) (IBL, USA) according to the manufacturer's instructions. The concentration of GLP-1 and CCK was expressed as pg/mL. The GLP-1 (IBL-RE53121, IBL, USA) and CCK (MM-0028M1) concentrations in the cell culture supernatant was calculated *via* ELISA. The concentration of GLP-1 and CCK was expressed as pmol/L. The plates were read at 450 nm using a plate reader.

### Western blot

The following primary antibodies were obtained from Santa Cruz Biotechnology (USA): GPR120 (1:1,000; sc-390752), PKC (1:1,000; sc-80), PLCβ1 (1:1,000; sc-5291), and IP3R (1:1,000; sc-271,197). Protein extraction and western blot (WB) were performed as previously described (17). Polyvinylidene difluoride (PVDF) (Bio-Rad, CA) membranes were incubated with primary antibodies overnight at 4 °C and then washed extensively. The membranes were incubated with HRP-labeled secondary antibody (1: 2,000; Goat anti-mouse secondary antibody, ABclonal, China) for 2 h at room temperature. Staining was performed using the Tanon High-sig ECL Western Blot Substrate kit (Pierce, USA) and visualized using an Odyssey infrared imaging system (Li-COR Biosciences, USA). β-actin expression was an internal quantitative control. The band densities were quantified using ImageJ (National Institutes of Health, USA).

### Immunofluorescence

Treated STC-1 cells grown on coverslips were fixed in 4% paraformaldehyde and incubated with the primary antibody GRP120 (1:100; AF5219, Affinity Biosciences, China) and IP3R (1:100) overnight at 4 °C, followed by washing with PBST three times (5 min each) and staining with fluorophore-conjugated goat anti-rabbit secondary antibody (Alexa Fluor 488 nm, Invitrogen, USA) and goat anti-mouse secondary antibody (Alexa Fluor 594 nm) for 2 h at room temperature in the dark. After washing three times with PBST, the cells were incubated with the appropriate secondary antibody in the dark at room temperature for 2 h. The cell nuclei were stained with DAPI (Sigma-Aldrich, USA) in the dark before the end of the procedure. The cells were then scanned using a laser scanning confocal microscope (Zeiss, Germany) to capture fluorescent images.

### Statistical analyses

The data were expressed as means ± SEM and were analyzed using IBM SPSS 23.0 Statistics (IBM, USA). Data normality was assessed. One-way analysis of variance (ANOVA) was used to identify significant differences among multiple groups. The Dunnett's *t*-test was used to identify significant differences between the two groups. Values of *P* < 0.05 was considered statistically significant.

## Results

### RS decreased feed intake of Hu sheep

**Figure 1:**
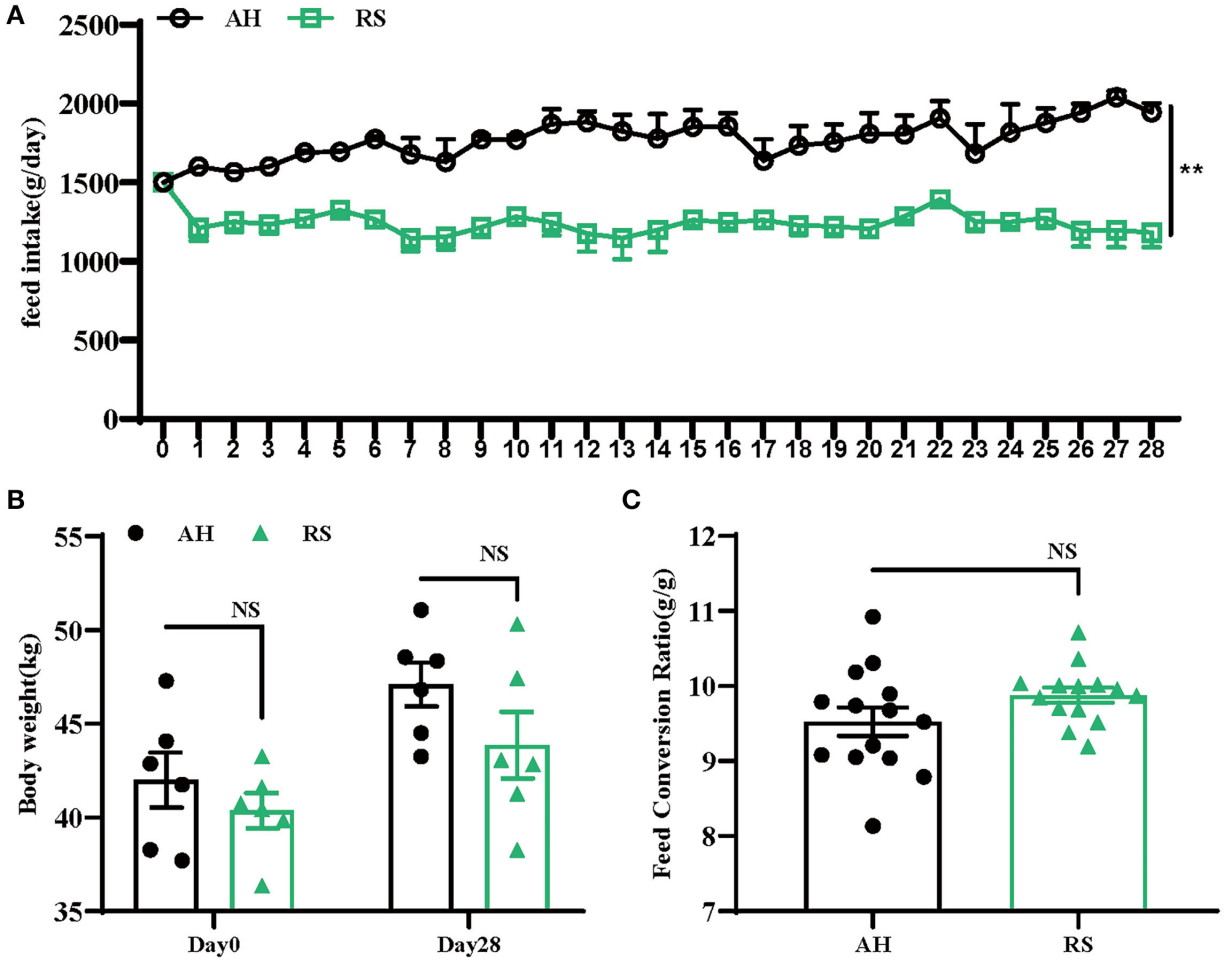
Effects of rice straw on growth performance of Hu Sheep: **(A)** Daily feed intake of Hu sheep; **(B)** Changes of body weight of Hu sheep during the experiment; **(C)** Change of feed conversion ratio (FCR) of Hu sheep during the experiment. Data are presented as the means ± SEM (*n* = 6). **P < 0.01 vs. AH group.

RS reduced the feed intake of Hu sheep during the experiment (*P* < 0.01) (Figure 1A). However, there were no significant differences in body weight (*P* = 0.412, *P* = 0.113) or FCR (*P* = 0.990) between the two groups (Figures 1B,C).

### RS induced metabolite changes in Hu sheep plasma

**Figure 2:**
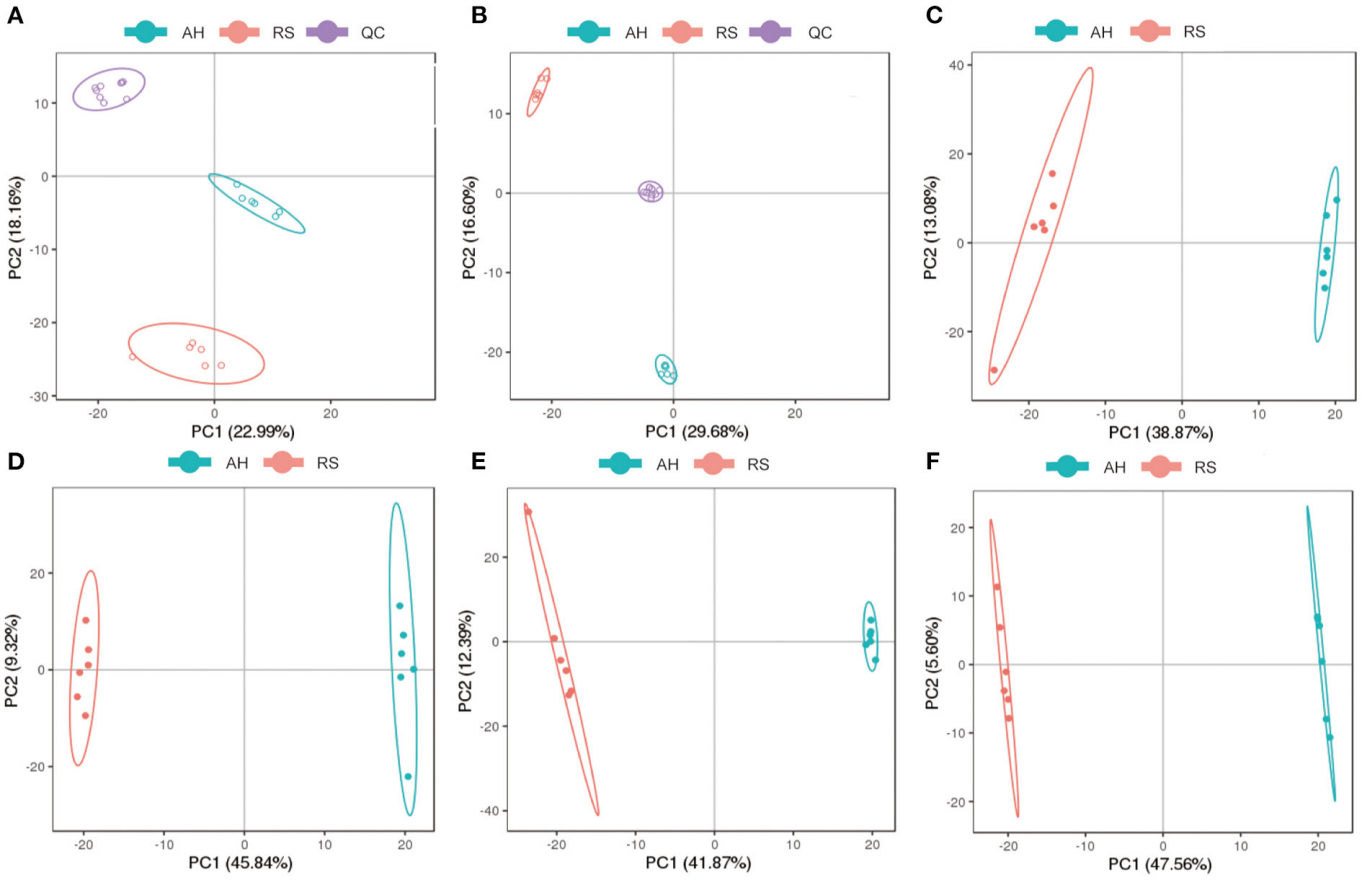
Analysis of differential metabolites: **(A,B)** Principal component analysis of QC samples in positive and negative ion models, respectively; **(C,D)** The PCA score map in positive and negative ion models, respectively; **(E,F)** The PLS-DA score map in positive and negative ion models, respectively. Blue dots represent AH group and red dots represent RS group.

**Figure 3:**
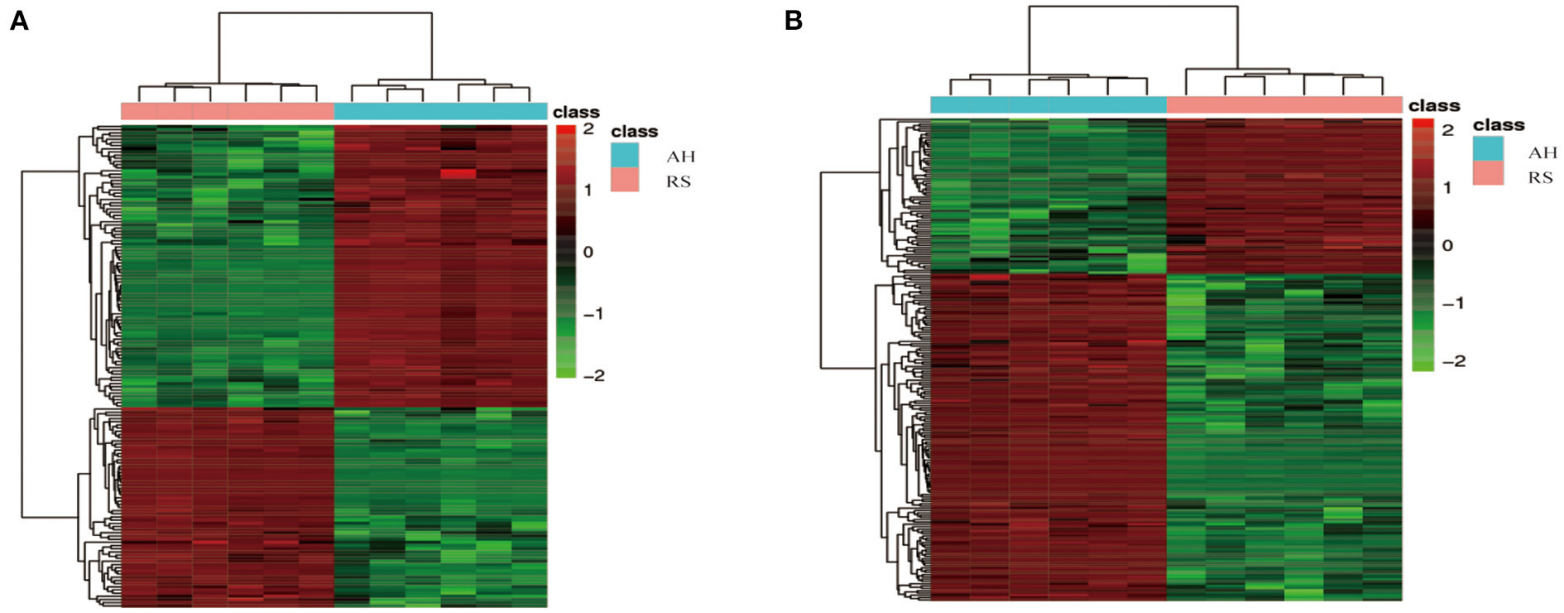
Cluster analysis of differential metabolites: **(A)** 89 significantly up-regulated and 63 significantly down-regulated differential metabolites in positive ion mode; **(B)** 78 significantly up-regulated and 133 significantly down-regulated differential metabolites in negative ion modes. The horizontal axis is the sample, and the vertical axis is the differential metabolite. Red, positive correlation; Green, negative correlation.

Using UPLC-MS/MS assays, principal component analysis score plots showed that the quality control samples were clustered tightly (Figures 2A–D). To gain a better understanding of the metabolism differences between the two experimental groups, PLS-DA was performed to construct an analytic model to discriminate between the respective samples (Figures 2E,F). Based on the cut-off values of VIP (> 1), fold change (≥ 2 or ≤ 0.5), and *P* (< 0.05), 152 positive ion mode and 211 negative ion mode significantly different metabolites were screened out as potential metabolic biomarkers (Supplementary Figure S1). To evaluate their plausibility, cluster analysis was performed to identify their differential abundance patterns in the plasma samples. Heat maps showed that 89 differentially changed ions were significantly upregulated, and 63 were significantly downregulated in positive ion mode (Figure 3A). In negative ion mode, 78 differential ions were upregulated, and 133 were downregulated (Figure 3B).

### RS induced changes in the lipid metabolism pathway in Hu sheep

**Figure 4:**
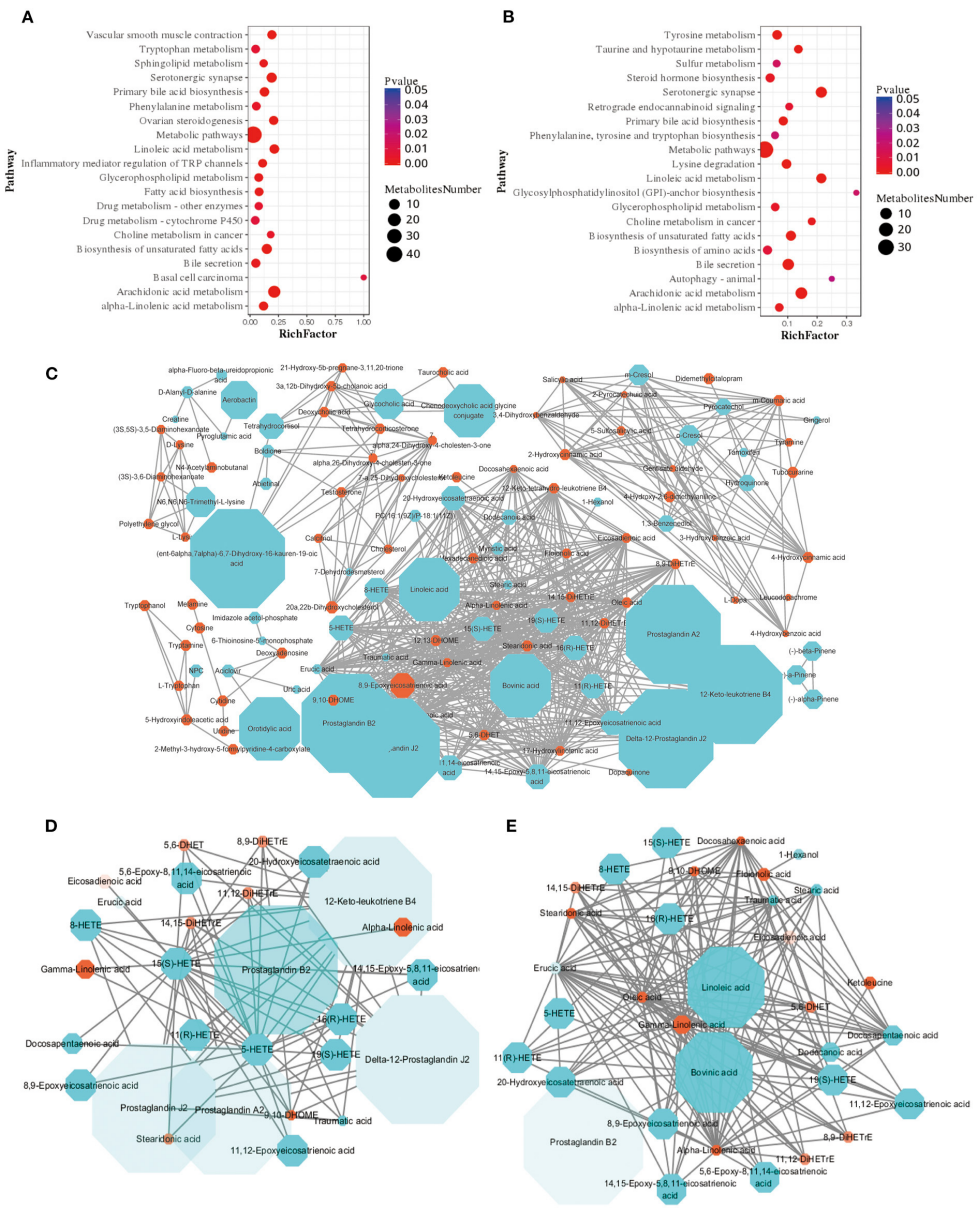
Enrichment analysis and metabolic network of differential metabolites: **(A,B)** Pathway enrichment analysis of differential metabolites in positive and negative ion models, respectively. **(C)** A fully connected network of all the differential metabolites; **(D)** Arachidonic acid network; **(E)** Linoleic acid and alpha-linolenic acid network. Blue dots indicate fold change (FC) ≥ 2 and orange dots indicate FC ≤ 0.5, the shape of the dots increases with the increase of FC, and the color transparency decreases with the decrease of *P* value.

Further analyse the metabolic processes of the differential metabolites in the plasma samples of the two groups, compared with AH group, the differential metabolites in RS group were mainly enriched in the arachidonic acid metabolism, biosynthesis of unsaturated fatty acids, linoleic acid metabolism, and alpha-linolenic acid metabolism pathways (Figures 4A,B), the core differential metabolites of RS group were SA, linoleic acid, and bovinic acid (Figures 4C–E). Moreover, compared with AH group, monounsaturated fatty acids (MUFAs) (*P* < 0.05), saturated fatty acids (SFAs) (*P* < 0.05), and LCFAs (*P* < 0.01), especially SA (*P* < 0.01), contents in the RS group plasma samples were significantly elevated (Supplementary Table S3).

### RS promotes satiety factor expression and secretion

**Figure 5:**
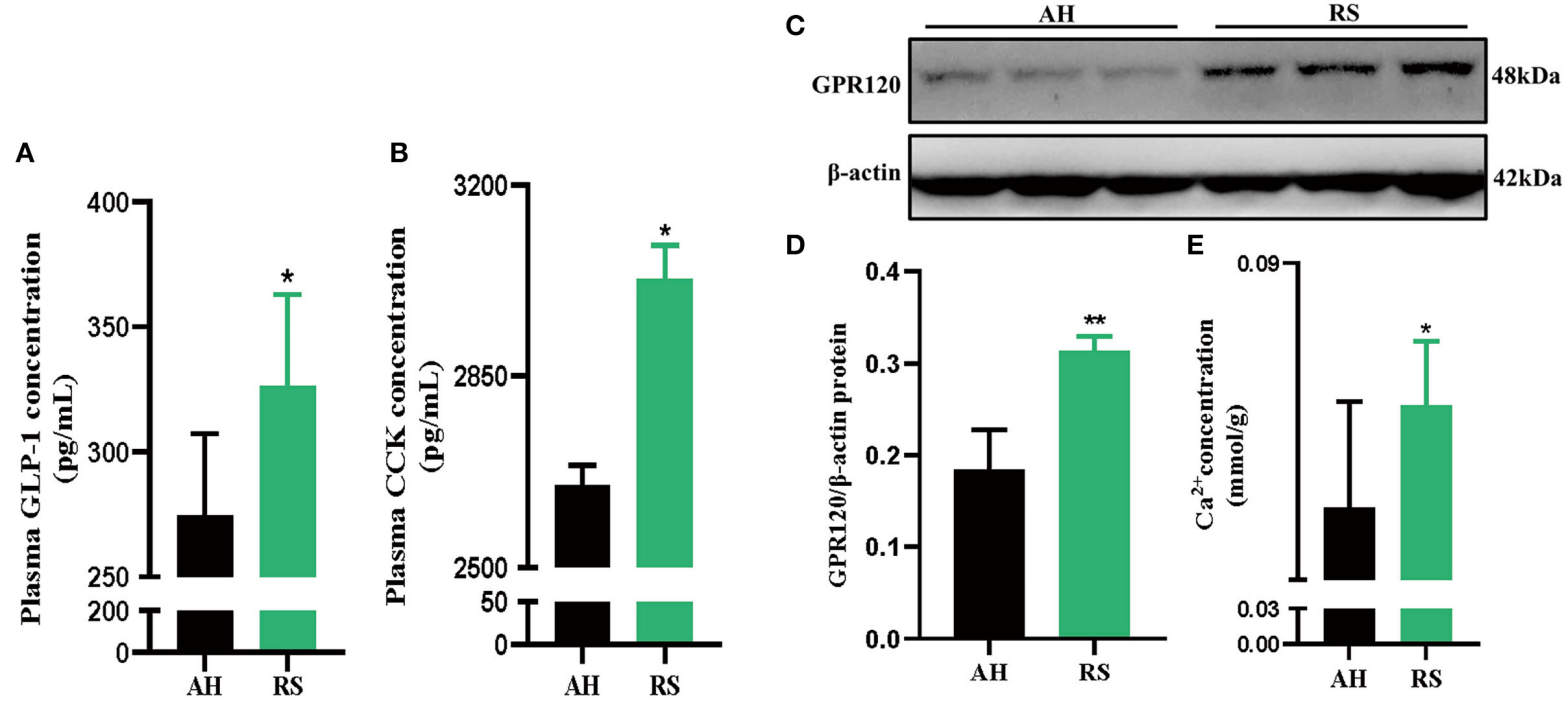
Effect of Rice Straw on Satiety appetite factor of Hu Sheep: **(A,B)** The secretion of GLP-1 and CCK in the plasma of Hu sheep; **(C,D)** GPR120 protein expression in Hu sheep jejunum; **(E)** The con-centration of Ca^2+^ in the jejunum of Hu sheep. Data are presented as the means ± SEM (*n* = 6). *P < 0.05, **P < 0.01 vs. AH group.

RS increased the levels of the satiety hormones GLP-1 (*P* < 0.05) and CCK (*P* < 0.05) in Hu sheep plasma (Figures 5A,B). Moreover, GPR120 protein expression (*P* < 0.01) and Ca^2+^ concentration (*P* < 0.05) in jejunum tissue were increased (Figures 5C–E).

### SA promoted the secretion of GLP-1 and CCK in STC-1 cells by binding to GPR120

**Figure 6:**
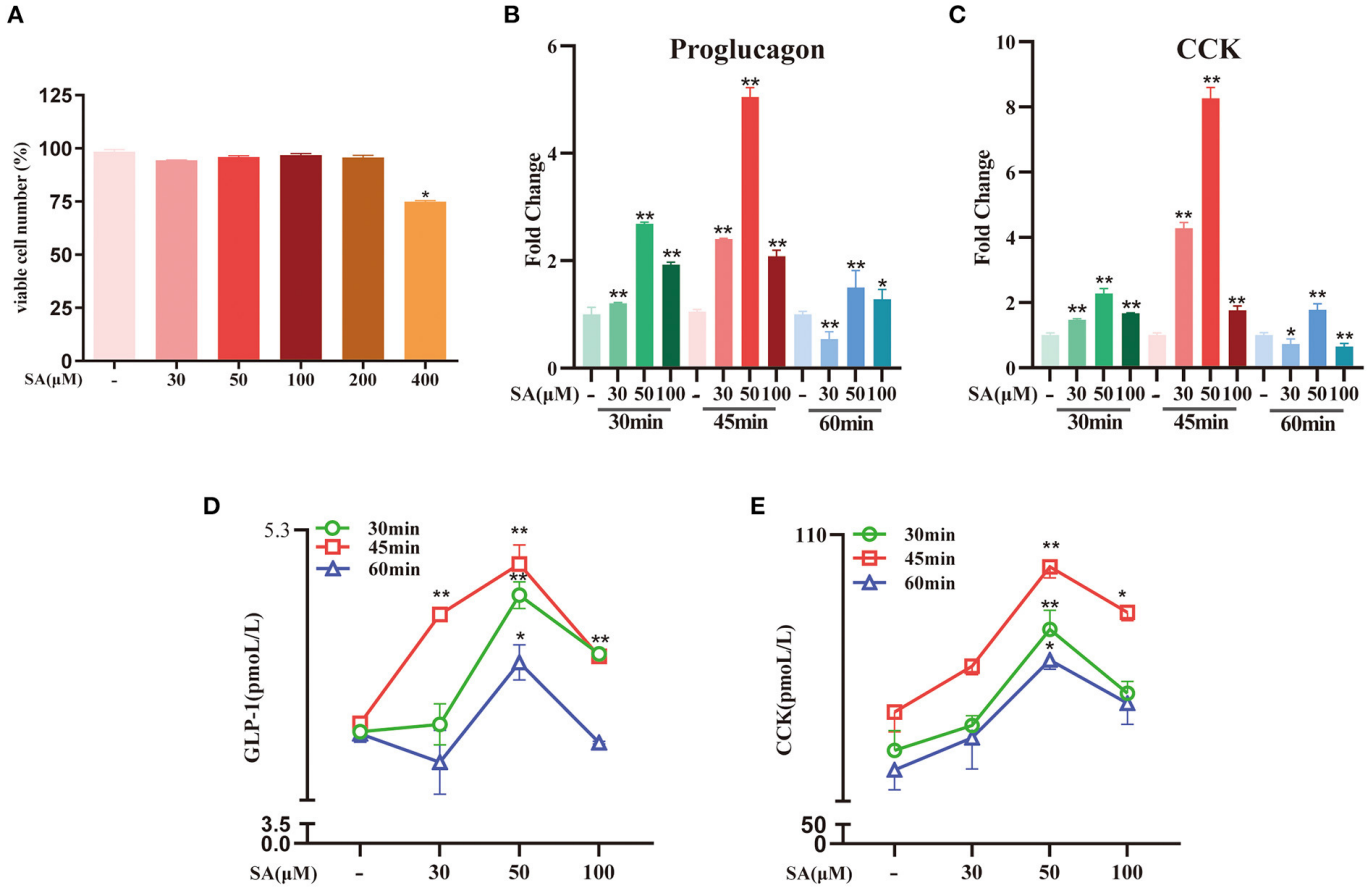
Effects of different concentrations of SA on secretion of GLP-1 and CCK in STC-1 cells: **(A)** CCK-8 assays were per-formed to estimate STC-1 cell vi-ability; **(B,C)** The expressions of proglucagon mRNA and CCK mRNA in STC-1 cells were detected by RT-qPCR; **(D,E)** The secretion of GLP-1 and CCK in STC-1 cells supernatant was determined by ELISA. Data are presented as the means ± SEM (*n* = 3). *P < 0.05, **P < 0.01 vs. Control for 30 min, 45 min and 60 min, respectively.

**Figure 7:**
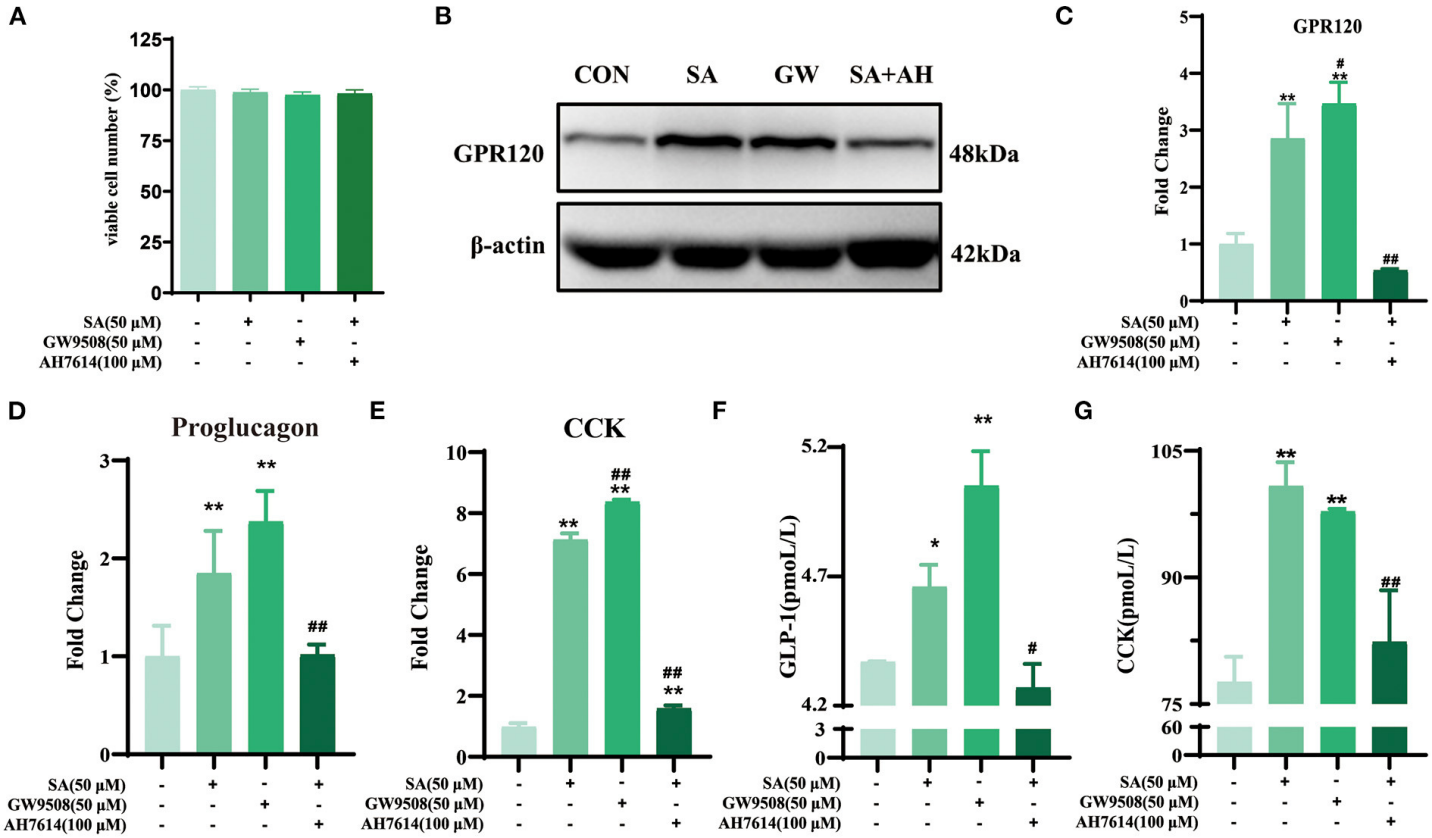
SA promoted the secretion of GLP-1 and CCK in STC-1 cells by binding to GPR120: **(A)** CCK-8 assays were per-formed to estimate STC-1 cell viability; **(B)** Protein expression of GPR120 in STC-1 cells determined by western blotting; **(C)** The expressions of GPR120 mRNA in STC-1 cells were detected by RT-qPCR; **(D,E)** The expressions of proglucagon mRNA and CCK mRNA in STC-1 cells were detected by RT-qPCR; **(F,G)** The secretion of GLP-1 and CCK in STC-1 cells supernatant was determined by ELISA. Data are presented as the means ± SEM (*n* = 3). *P < 0.05, **P < 0.01 vs. Control (SA 0 μM). ^#^*P* < 0.05, ^*##*^*P* < 0.01 vs. SA (50 μM).

The CCK-8 test results showed that only 400 μM of SA reduced STC-1 cell viability for 2 h (*P* < 0.05) (Figure 6A). Compared with the control group, SA increased the expression of proglucagon mRNA (*P* < 0.01) and CCK mRNA (*P* < 0.01) in STC-1 cells (Figures 6B,C). The secretion of GLP-1 (*P* < 0.01) and CCK (*P* < 0.01) peaked with 50 μM of SA for 45 min (Figures 6D,E). The cell viability of STC-1 was not significantly decreased after incubation with GW9508 or AH7614 for 45 min and SA for 45 min, respectively (Figure 7A). SA and the GPR120 agonist GW9508 had similar effects, significantly increasing the mRNA (*P* < 0.01; *P* < 0.01) and protein expression of GPR120 in STC-1 cells compared with the control group. AH7614, a GPR120 inhibitor, blocked the upregulation of SA in GPR120 mRNA (*P* < 0.01) and protein expression in STC-1 cells (Figures 7B,C). Further detection of CCK and proglucagon mRNA expression and secretion in cells showed that AH7614 blocked the promotional effect of SA on GLP-1 (*P* < 0.01; *P* < 0.05) and CCK (*P* < 0.01; *P* < 0.01) (Figures 7D–G).

### SA promoted the secretion of GLP-1 and CCK in STC-1 cells by activating the PLCβ/PKC/IP3R signaling pathway

**Figure 8:**
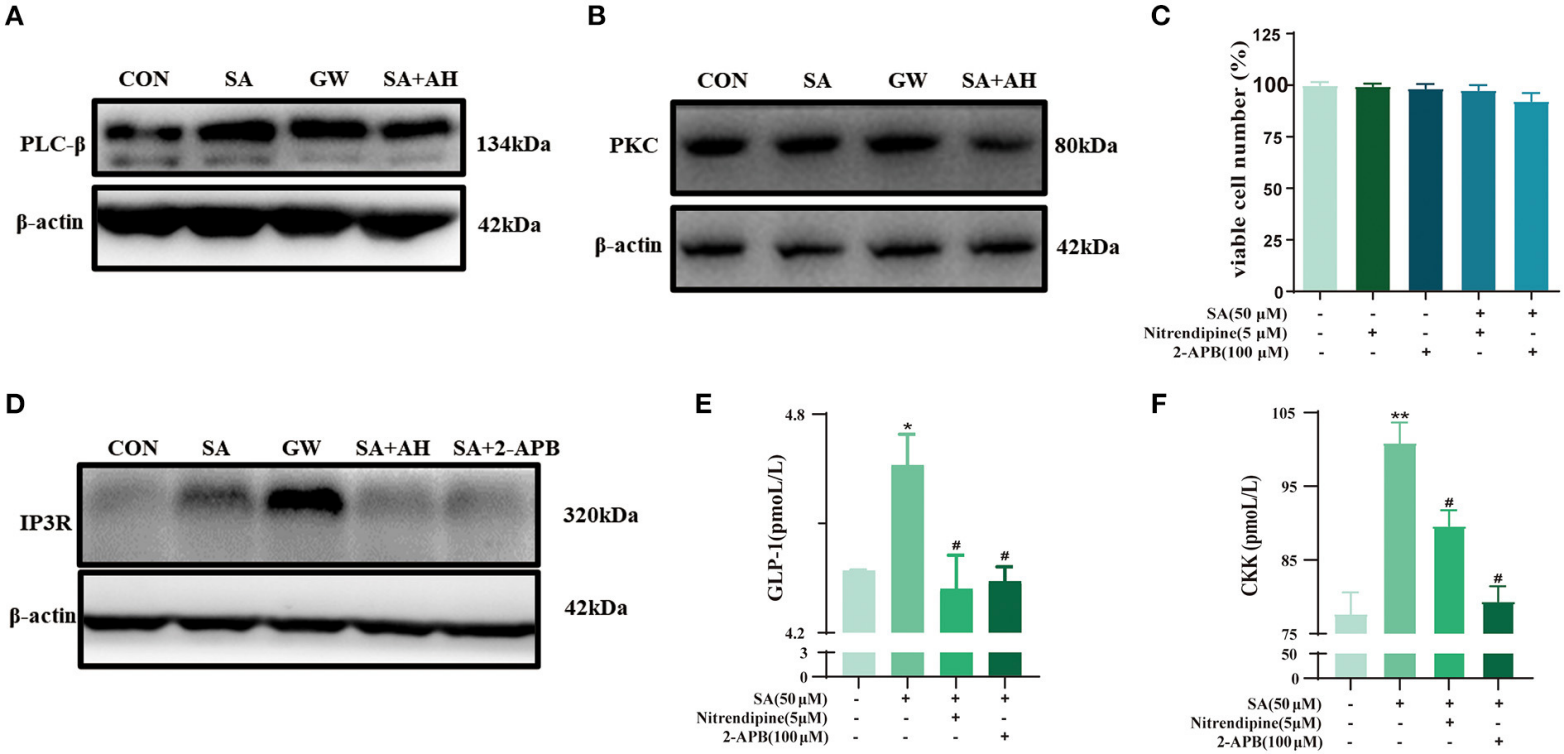
SA promoted the secretion of GLP-1 and CCK in STC-1 cells by binding to GPR120: **(A,B)** Protein expression of PLCβ and PKC in STC-1 cells determined by western blotting; **(C)** CCK-8 assays were per-formed to estimate STC-1 cell viability; **(D)** Protein expression of IP3R in STC-1 cells determined by western blotting; **(E,F)** The secretion of GLP-1 and CCK in STC-1 cells supernatant was determined by ELISA. Data are presented as the means ± SEM (*n* = 3). *P < 0.05, **P < 0.01 vs. Control (SA 0 μM). ^#^*P* < 0.05 vs. SA (50 μM).

**Figure 9:**
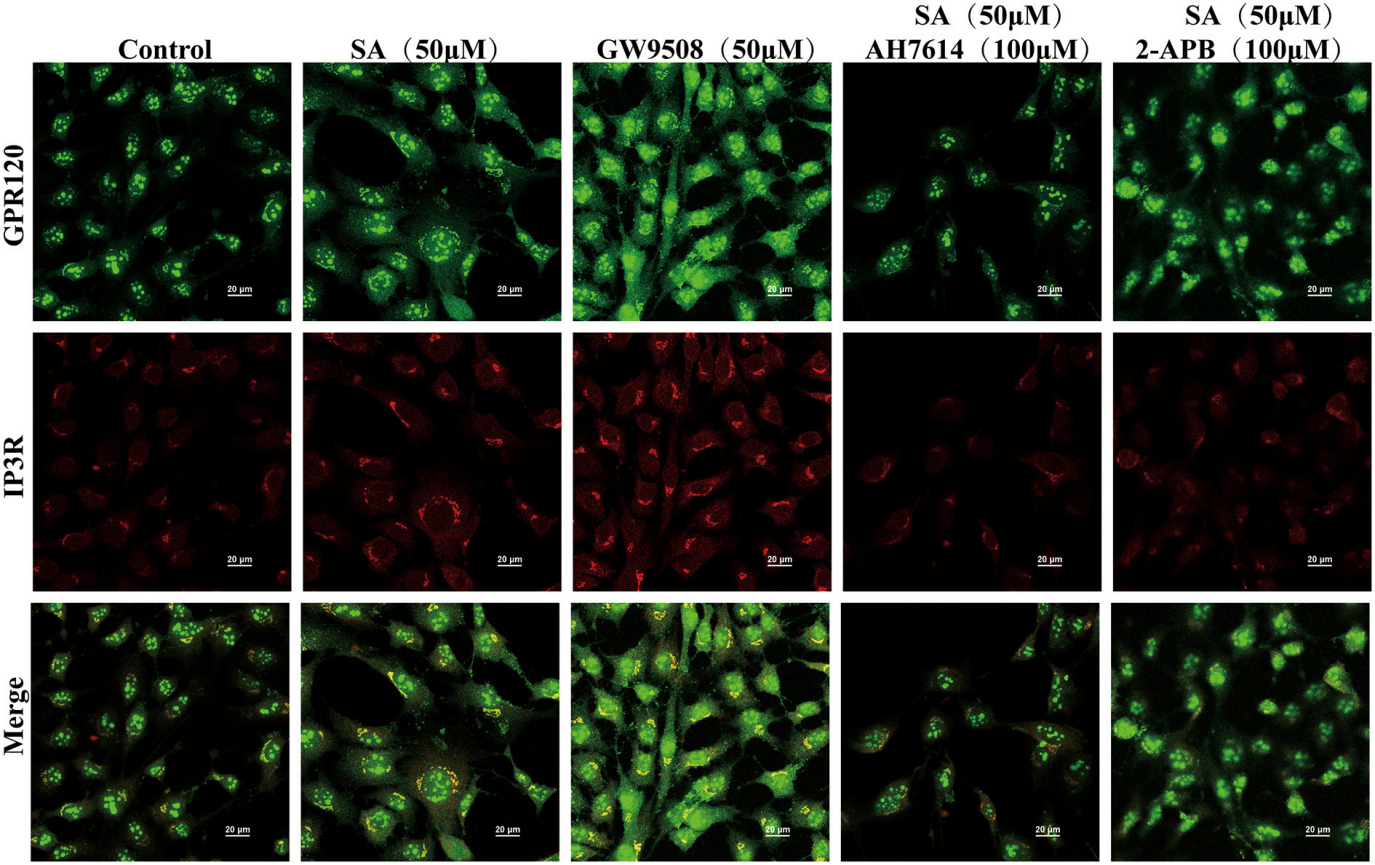
Immunofluorescence analysis examined the expression of GPR120 and IP3R in STC-1 cells. The scale bar represents 20 μm. Green, GPR120; Red, IP3R.

To explore the GPR120-mediated signaling channels related to intracellular Ca^2+^, western blotting was performed. SA increased PLCβ and PKC protein expression. This effect was reversed by AH7614 (Figures 8A,B). The cell viability of STC-1 was not significantly decreased after incubation with nitrendipine or 2-APB for 15 min and SA for 45 min, respectively (Figure 8C). SA increased IP3R protein expression, this effect was reversed by AH7614 and 2-APB (Figure 8D). Immunofluorescence results also con-firmed that the protein expression of GPR120 and IP3R in STC-1 cells was increased in SA or GW9508 treatment groups, while the protein expression of GPR120 and IP3R was significantly decreased in AH7614 treatment group, and the protein expression of IP3R was significantly decreased in 2-APB treatment group (Figure 9). Moreover, both nitrendipine and 2-APB blocked the promotive effect of SA on GLP-1 (*P* < 0.05; *P* < 0.05) and CCK (*P* < 0.05; *P* < 0.05) secretion (Figures 8E,F).

## Discussion

Appetite is an instinctive desire necessary to maintain organic life, and different factors can affect appetite and alter feed intake. Exogenous CCK has been shown to reduce food intake in a dose-dependent manner in various mammals, whereas normal postprandial circulating CCK levels have been shown to reduce meal size and promote satiety (18). Study has confirmed that peripherally administered physiological doses of GLP-1 can reduce meal size in rodents and humans, and that postprandial plasma concentrations of GLP-1 can cause satiety in rodents and humans (19). The combined injection of GLP-1 and CCK (20) or coadministration of a glucagon-like peptile-1 receptor (GLP-1R) agonist with a cholecystokinin Receptor-1 (CCKR1) agonist (21–23) can inhibit appetite and reduce body weight. Our results were similar, we found that Hu sheep fed with RS exhibited significantly reduced feed intake, which was associated with high CCK and GLP-1 levels in their blood. The interaction and synergy between CCK and GLP-1 will be further studied in the future.

A variety of fatty acids in ruminants mainly come from fat metabolism in the rumen, and fat mainly comes from feed and grain (24). Intermediate metabolites generated from feed in the rumen enter the intestine through lipase degradation and hydrogenation by rumen microorganisms, are absorbed and then enter the various tissues (25). Study has investigated the feed conversion rate of ruminants by metabolomics and targeted lipidomics of multiple tissues. The results confirmed that the α-linolenic acid pathway was significantly down-regulated while oleic acid and SA were significantly increased in steers fed high-concentrate diets with low ADG (26). In order to investigate the effects of metabolic intermediates produced by RS on feed intake reduction and satiation hormone release in Hu sheep, we conducted a comparative metabolomics study on blood samples of Hu sheep fed AH and RS. The results also showed that α-linolenic acid pathway was significantly down-regulated, while oleic acid and SA were significantly increased in the RS group. Linoleic acid has been shown to inhibit short-term food intake in rats (27). Central injections of oleic acid or excess long-chain saturated fatty acids have been shown to reduce food intake and impair appetite in both human and rodent studies (28). SFAs was found to be negatively correlated with appetite and circulating GLP-1 levels in humans (29). Meanwhile, the significantly up-regulated pathways of arachidonic acid metabolism and linoleic acid metabolism in the RS group and the hydrogenation and other pathways of core differential metabolites ultimately pointed to the same saturated long-chain fatty acid: SA. And our results indicated that the decreased feed intake of RS fed Hu sheep may be related to the activation of lipid metabolism-related pathways and plasma LCFAs (especially SA) concentration.

Based on the above research background and experimental results, we focused on the role and molecular mechanism of SA in the regulation of appetite in ruminants. GPCRs, which have been proven to recognize endogenous and dietary FFAs, constitute the most numerous and diverse family of cell surface receptors involved in the regulation of various physiological processes, including GPR40, GPR43, GPR41, and GPR120 (30). GPR120 can be activated by saturated fatty acids with chain length C14 to C18 and unsaturated fatty acids with chain length C16 to C22 (31) and plays an important role in various physiological processes involved in LCFAs. As an LCFA sensor, GPR120 plays an important role in various physiological processes involved in LCFAs and is one of the key factors in the mechanism of appetite regulation. In our study, we found that RS activated the expression of GPR120 in the jejunum by increasing the SA content. Previous studies on LCFA–GPR120-mediated signal transduction has reported that GPR120 induces a significant intracellular Ca^2+^ influx under the influence of FFAs. For example, activation of GPR120 by agonists such as LCFAs or synthetic ligands can increase intracellular Ca^2+^ levels in cells expressing GPR120 from human or mouse, in addition, GPR120 activation increases plasma insulin and GLP-1 levels in mice receiving oral ALA (32). After activation of GPR120 by LCFAs and other agonists, the subsequent intracellular Ca^2+^ peak induces the release of GLP-1 (33) and CCK (34). Our *in vivo* results are similar, showing that the high LCFA levels in the blood of Hu sheep fed with RS promoted the secretion of CCK and GLP-1 by mediating the GPR120/Ca^2+^ signaling pathway in the jejunum, ultimately leading to reduced feed intake.

The question that arises is how LCFAs, especially SA, regulate satiety hormones. Monosaccharides, fatty acids, aromatic amino acids, peptide-like compounds, and bitter taste enhancers have been shown to induce the secretion of CCK and GLP-1 by STC-1 cells (35). FFAs can induce GLP-1 and CCK secretion through GPCRs, such as GPR40 and GPR120, which have been shown to severely impair LCFA-induced hormone release in GPR120-knockout mice, whereas blocking GPR40 has been shown to have little effect on the ability to perceive fatty acids (36). Our *in vitro* study also confirmed that SA plays a role in promoting GLP-1 and CCK by binding to GPR120.

FFAs induce GLP-1 and CCK secretion through GPR120 in intestinal endocrine cells *via* a signal transduction pathway that involves an increase in intracellular Ca^2+^ concentrations. However, the origin and transduction mechanism of the signal remain unclear. Our experimental results showed that blocking the influx of Ca^2+^ reduced GLP-1 and CCK secretion, suggesting that the promotion of GLP-1 and CCK secretion by SA requires an increase in cell Ca^2+^ concentrations. Ca^2+^ mobilization is a highly controlled process in eukaryotes and one of the main methods of regulating excitable cells (37). PLC responds to stimuli from growth factors, neurotransmitters, hormones, and many other extracellular signals and hydrolyses the plasma membrane lipid phosphatidylinositol-4, 5-diphosphate (PIP2) to diacylglycerol (DAG) and inositol-1,4,5-triphosphate (IP3). DAG and IP3 are important secondary messengers of PKC activation in response to many cellular signals. They stimulate intracellular Ca^2+^ release and activate PKC to regulate various physiological activities (38). IP3 is an important messenger of Ca^2+^ signals downstream of GPCRs and receptor tyrosine kinases, and IP3R is one of the main Ca^2+^ release channels in the endoplasmic reticulum (39). To explore the mechanism of intracellular Ca^2+^ signal transduction, we investigated the proteins affecting the Ca^2+^-related pathways and found that SA binding to GPR120 activated its downstream PLCβ and PKC. GPR120 activation has been reported to stimulate pancreatic polypeptide secretion via the PLC/Ca^2+^ signaling pathway. However, its effect is diminished in obese mice fed a high-fat diet (40). Our study also confirms the importance of IP3R in mediating signals from GPR120 to activate downstream Ca^2+^. The high SA levels in the blood of Hu sheep fed an RS-based diet can activate the PLCβ/PKC/IP3R signaling pathway by combining with GPR120, thereby promoting the secretion of GLP-1 and CCK, which leads to reduced feed intake.

## Conclusions

**Figure 10:**
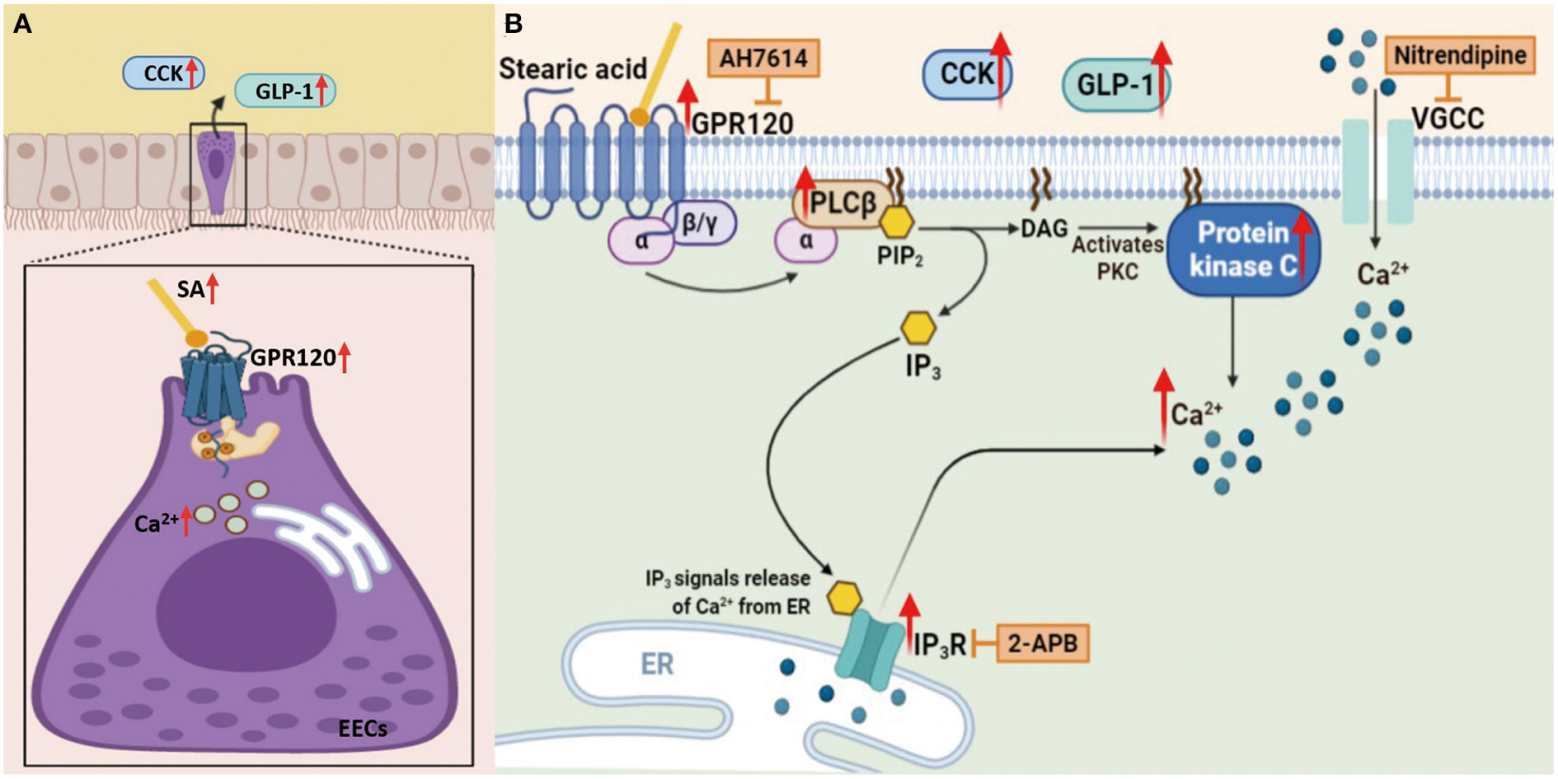
Schematic representation of SA induces CCK and GLP-1 upregulation *via* GPR120/PLC-β, leading to reduced appetite in Hu sheep fed with rice straw (Charting with BioRender.com software): **(A)** *In vivo* results showed that an RS-based diet increased the content of SA, then the subsequent activation of the LCFA receptor GPR120 caused a significant influx of Ca^2+^ and a significant secretion of the downstream satiety hormones GLP-1 and CCK, which inhibited appetite and reduced feed intake in Hu sheep; **(B)** *In vitro* mechanism studies showed that SA promotes GLP-1 and CCK secretion by activating GPR120-mediated downstream PKC and IP3R signaling pathways of PLCβ.

RS is one of the most commonly used types of roughage in animal husbandry. Feeding ruminants with RS alone reduces feed intake, which affects livestock production efficiency. Our results showed that an RS-based diet activated multiple metabolic pathways and significantly increased the content of SA, their metabolic end product. The subsequent activation of the LCFA receptor GPR120 caused a significant influx of Ca^2+^ and a significant secretion of the downstream satiety hormones GLP-1 and CCK, which inhibited appetite and reduced feed intake in Hu sheep (Figure 10A). *In vitro* mechanism studies showed that SA promotes GLP-1 and CCK secretion by activating GPR120-mediated downstream PKC and IP3R signaling pathways of PLCβ (Figure 10B).

## Data availability statement

The raw data supporting the conclusions of this article will be made available by the authors, without undue reservation.

## Ethics statement

The animal study was reviewed and approved by the Ethics Committee of Nanjing Agricultural University.

## Author contributions

Conceptualization and methodology: XC and XN. Software: XC, XN, and HW. Validation, formal analysis, investigation, and visualization: XC, HW, and SY. Resources and writing-review and editing: XN and YZ. Data curation and writing-original draft preparation: XC. Supervision: XC and YZ. Project administration and funding acquisition: YZ. All authors contributed to the article and approved the submitted version.

## Funding

This research was funded by the thirteen-five national key research and development plan (2017YFD0500505) and the Priority Academic Program Development (PAPD) of Jiangsu Higher Education Institutions.

## Conflict of interest

The authors declare that the research was conducted in the absence of any commercial or financial relationships that could be construed as a potential conflict of interest.

## Publisher's note

All claims expressed in this article are solely those of the authors and do not necessarily represent those of their affiliated organizations, or those of the publisher, the editors and the reviewers. Any product that may be evaluated in this article, or claim that may be made by its manufacturer, is not guaranteed or endorsed by the publisher.
