# Supplementary material for: Stearic acid induces CCK and GLP-1 upregulation via GPR120/PLC-β, leading to reduced appetite in Hu sheep fed with rice straw
Source: Front Vet Sci. 2022 Sep 2;9:948074. doi: 10.3389/fvets.2022.948074 (PMC9478758; doi:10.3389/fvets.2022.948074)
Supplement: Supplementary file 1 [file Data_Sheet_1.docx]

Supplementary Material

# Supplementary Tables

**Table S1.** Composition and nutrient levels of basal diets (air-dry basis) %.

| **Items** | **Alfalfa hay** | **Rice straw** |
| --- | --- | --- |
| Ingredients |  |  |
| Corn | 30.0 | 33.0 |
| Soybean meal | 0 | 18.5 |
| Rice straw | 0 | 40.0 |
| Alfalfa hay | 40.0 | 0 |
| Wheat bran | 24.5 | 5.0 |
| Zeolite powder | 2.5 | 0 |
| Limestone | 0 | 0.5 |
| baking soda | 0.5 | 0.5 |
| Premix ^1^ | 2.5 | 2.5 |
| Total | 100.0 | 100.0 |
| Nutrient levels ^2^ |  |  |
| ME/（MJ/kg） | 19.63 | 19.42 |
| CP | 12.80 | 12.82 |
| EE | 2.51 | 2.107 |
| NDF | 54.62 | 69.74 |
| ADF | 42.37 | 42.20 |
| Ash | 7.30 | 7.60 |
| Ca | 0.53 | 0.49 |
| TP | 0.47 | 0.32 |

^1^ The premix provided the following per kg of diets: VA 50 000 IU, B1 25 mg, VB2 95 mg, VB5 40 mg, VB6 25 mg, VB12 0.38 mg, VD3 17 500 IU, VE 1100 IU, VK3 12.5 mg, biotin 5 mg, folic acid 32.5 mg, *D*-pantothenic acid 300 mg, nicotinic acid 250 mg, Cu (as copper sulfate) 375 mg, Fe (as ferrous sulfate) 1750 mg, Mn (as manganese sulfate) 625 mg, Zn (as zinc sulfate) 2000 mg, I (as potassium iodide) 3.50 mg, Se (as sodium selenite) 3.75 mg.

^2^ ME was a calculated value, while the others were measured values.

**Table S2.** The primer sequences of the genes

| **Gene** | **Accession No.** | **Primers sequence（5’-3’）** | **Orientation** | **Product size** |
| --- | --- | --- | --- | --- |
| *GAPDH* | XM_017321385.2 | ATGACATCAAGAAGGTGGTG | Forward  Reverse | 177 bp |
|  |  | CATACCAGGAAATGAGCTTG |  |  |
| *GPR120* | NM_181748.2 | CTGGGGCTCATCTTTGTCGT | Forward  Reverse | 155 bp |
|  |  | ACGACGAGCACTAGAGGGAT |  |  |
| *proglucagon* | NM_008100.4 | ATTGCCAAACGTCATGATGA | Forward  Reverse | 150bp |
|  |  | GGCGACTTCTTCTGGGAAGT |  |  |
| *CCK* | NM_001284508.2 | CCAATTTTTCCTGCCCGCAT | Forward  Reverse | 74bp |
|  |  | AGAAGGAGCAGTCAAGCCAAA |  |  |

**Table S3.** Fatty acids concentrations in plasma in Hu sheep after feeding different diets.

| **Items (mg/L)** | **AH** | **RS** | ***P* value** |
| --- | --- | --- | --- |
| c10:0 | 0.16±0.01 | 0.12±0.01 | 0.816 |
| c14:0 | 0.82±0.06 | 0.8010±0.03 | 0.981 |
| c15:0 | 0.62±0.06 | 0.75±0.06 | 0.852 |
| c16:0 | 18.54±0.13 | 19.70±0.94 | 0.092 |
| c16:1 | 1.36±0.12 | 1.31±0.07 | 0.939 |
| c17:0 | 0.95±0.01 | 0.93±0.01 | 0.973 |
| c17:1 | 0.45±0.01 | 0.56±0.02 | 0.871 |
| c18:0 | 19.92±0.72 | 22.64±0.86 | 0.0004 |
| c18:1n9t | 0.78±0.07 | 0.76±0.05 | 0.98 |
| c18:1n9c | 17.33±0.13 | 22.80±1.172 | 0.001 |
| c18:2n6c | 21.35±1.08 | 15.78±1.01 | 0.001 |
| c20:0 | 0.14±0.01 | 0.18±0.03 | 0.776 |
| c18:3n6 a | 0.42±0.01 | 0.32±0.01 | 0.877 |
| c18:3n3 r | 1.31±0.14 | 0.41±0.03 | 0.001 |
| c22:0 | 0.27±0.04 | 0.34±0.05 | 0.597 |
| c20:3n6 | 0.30±0.01 | 0.30±0.01 | 0.953 |
| c23:0 | 2.52±0.29 | 2.81±0.11 | 0.061 |
| c20:5n3 E | 0.14±0.01 | 0.25±0.01 | 0.815 |
| c22:6 D | 0.25±0.02 | 0.23±0.02 | 0.986 |
| Monounsaturated fatty acids (MUFAs) | 19.923±0.08 | 25.43±1.14 | 0.04 |
| Polyunsaturated fatty acids (PUFAs) | 23.64±1.20 | 17.04±1.03 | 0.052 |
| Unsaturated fatty acids (UFAs) | 43.56±1.12 | 42.47±0.11 | 0.537 |
| Saturated fatty acids (SFAs) | 43.63±0.91 | 47.98±0.03 | 0.041 |
| Long chain fatty acids (LCFAs) | 87.20±0.21 | 90.44±0.13 | 0.008 |

AH, alfalfa hay group; RS, rice straw group; Values are shown as the means ± SEM, n=6/group.

#
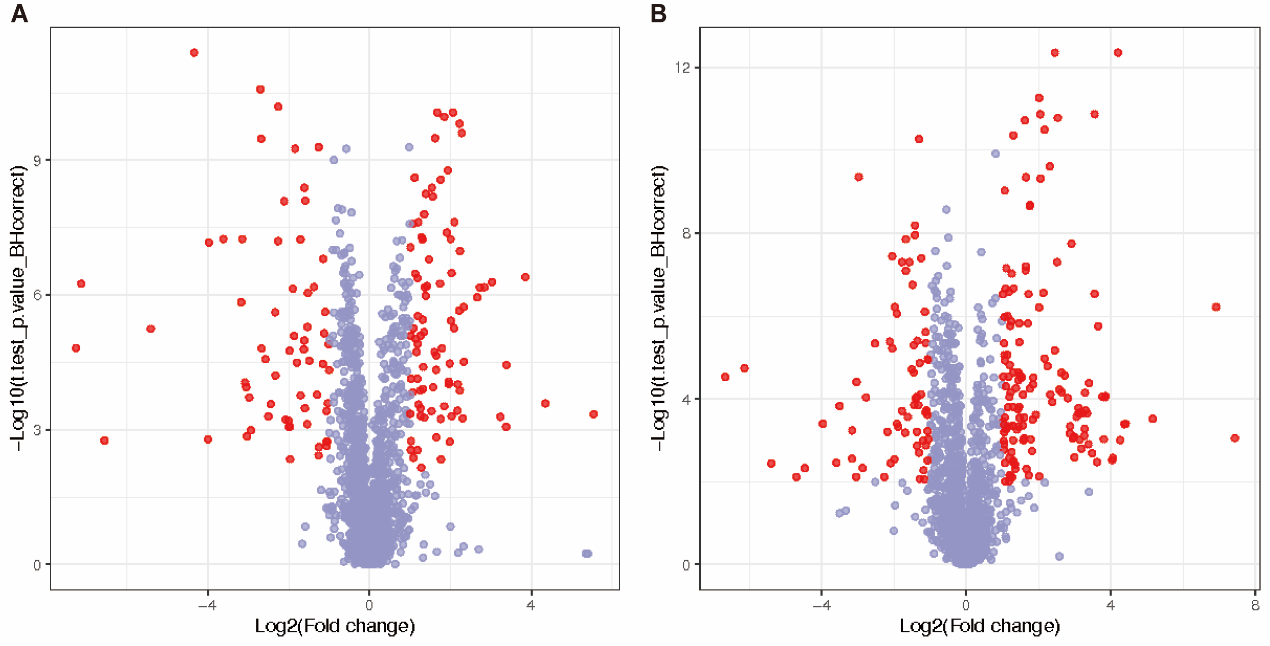
Supplementary Figures

**Supplementary Figure 1.** Volcano plot of differential metabolites. The red dots represent the differential metabolites, and the X-axis and Y-axis are log2 (FC) value and -log10 (P) value respectively: (A) 152 differential metabolites were screened in positive ion mode; (B) 211 differential metabolites were screened in negative ion mode. The screening criteria were as follows: 1) VIP ≥ 1; 2) fold change ≥ 2 or ≤ 0.5; 3) *P* values < 0.01.
